# High tackle headache: implications of referee agreement for tackle height law change

**DOI:** 10.1136/bmjsem-2024-002347

**Published:** 2025-01-20

**Authors:** Ruth Leese, Ash Kolstad, Ricardo T Sant'Anna, Carly D McKay, Stephen W West

**Affiliations:** 1 Department for Health, University of Bath, Bath, UK; 2 Sport Injury Prevention Research Centre, Faculty of Kinesiology, University of Calgary, Calgary, Alberta, Canada; 3 Department of Sport & Physical Activity, Edge Hill University, Ormskirk, Lancashire, UK; 4 UK Collaborating Centre on Injury and Illness Prevention in Sport, University of Bath, Bath, UK; 5 Centre for Health, and Injury and Illness Prevention in Sport, University of Bath, Bath, UK

**Keywords:** Rugby, Injury, Prevention, Implementation

## Abstract

**Objectives:**

Rugby Union has a relatively high risk of injury. Early evidence suggests a benefit of lowering tackle height to reduce head and neck injuries, although concerns persist among stakeholders regarding implementation challenges. This study aimed to understand whether referees can reach the same conclusion regarding tackle height in a controlled environment (ie, video) and whether priming influenced these decisions.

**Methods:**

Forty-eight active referees completed a questionnaire based on high-tackle decision-making guidelines after watching tackles. Participants were randomly assigned one of two instructional videos containing a high or legal tackle to investigate the impact of priming on law interpretation.

**Results:**

The percent agreement regarding tackle height was 78.1% between participants, 62.7% between participants and an experienced analyst, and 74.0% between participants and a gold-standard referee. Mean intra-rater reliability when determining whether a tackle was high was substantial (percent agreement: 91.2%). For high tackles, 83% of participants agreed on the danger level, 57% on the contact location and 71% on the presence of mitigating factors. No significant effects of priming were observed. Inter-rater agreement among participants and the gold-standard referee was moderate for all items except danger and height, which showed strong agreement.

**Conclusion:**

These results suggest a need for improved referee training to support changes to the legal tackle height.

**WHAT IS ALREADY KNOWN ON THIS TOPIC**

- Changing the legal tackle height in community and youth rugby union has been identified as a potential strategy for reducing the risk of concussion.
- Referees provide the means for enforcement of such a law change and, as such, must be equipped to make appropriate and consistent decisions.

**WHAT THIS STUDY ADDS**

- This study reports the agreement (Gwet’s Agreement Coefficient (AC): 0.39 (0.23–0.55)) and consistency (Gwet’s AC: 0.85 (0.52–1.00)) between a sample of current referees under controlled video analysis conditions.
- This study also highlights potential limitations and implementation challenges which may contribute to the success of such tackle height law changes.

**HOW THIS STUDY MIGHT AFFECT RESEARCH, PRACTICE OR POLICY**

- This study suggests the need for greater training and resourcing for current referees to support the implementation of a tackle height intervention.
- This study also suggests the need for referee input on video analysis studies examining the tackle in rugby.

## Introduction

### Background

 Rugby Union (Rugby hereafter) is a popular sport with over 9.1 million participants across 123 countries.^1^ However, rugby carries a high injury risk compared with many other sports, which may be detrimental to the sport’s popularity and the retention and welfare of players. The tackle constitutes the primary event leading to injuries in rugby, comprising 43% of injuries in professional rugby, 59% (men) and 61% (women) of injuries in community Rugby, and between 55 and 71% of injuries in boys’ and girls’ youth rugby.^2^,^4^ Furthermore, with increased recognition and diagnosis of concussions, the rates and severity have increased, with most of these concussions occurring in the tackle.^5^

Lowering the legal tackle height has been identified as a promising strategy for reducing injuries and concussions in Rugby.^6 7^ Trials in South Africa and France lowering legal tackle heights (to the armpit and base of the sternum, respectively) reduced injuries and increased participation, prompting global adoption among select unions.^8^ By aligning these tackle height interventions with existing regulations, referees are better equipped to make consistent decisions, improving player safety and promoting the integrity of the game.

World Rugby^9^ Law 9.13 states, ‘A player must not tackle an opponent early, late or dangerously. Dangerous tackling includes, but is not limited to, tackling or attempting to tackle an opponent above the line of the shoulders even if the tackle starts below the line of the shoulders’. World Rugby^8^ defines a high tackle as an illegal tackle involving clear contact with the ball carrier’s (BC’s) head or neck, visible head movement from the tackle or the BC needing a head injury assessment. To support referee decision-making in circumstances of head contact, World Rugby has developed a framework that includes factors related to head contact, foul play, degree of danger, point of contact and mitigating circumstances. If mitigating factors exist (eg, the tackler makes a definite attempt to change the height to avoid the ball carrier’s head; online supplemental appendix A), consequences may be reduced by one level. The four levels of penalisation, from highest to lowest, are red card and penalty kick, yellow card and penalty kick, penalty kick only, and no penalty (play on).

The effectiveness of a policy change can be influenced by referee training and enforcement of such policy during gameplay. For injury prevention strategies to be successful, effective implementation strategies are required to ensure appropriate adherence and adoption of the interventions.^10 11^ However, the high demands placed on referees can induce fatigue that can subsequently impair their decision-making accuracy, particularly in the final minutes of matches, as demonstrated in other sports such as Rugby League^12^ and Association Football,^13^ highlighting the importance of proper training to mitigate the effects of fatigue. Lane *et al*^14^ developed a framework based on interviews with football referees, identifying three key themes that influence decision-making: accuracy-error, regulations and professionalism. These themes contribute to an ‘ideal decision-making’ model affected by individual, experiential and situational factors, highlighting the need to assess referees’ decision-making without situational influences. Despite not being available in most settings, video analysis is crucial for assessing referees’ decision-making in critical situations.^10^ This may underscore the importance of using video footage to evaluate referees’ application of laws like the new tackle height law.

Determining whether priming or bias may affect a referee’s decisions is also important. Priming refers to how introducing a stimulus (ie, a tackle) influences the response to a subsequent one.^15^ Within this context, it refers to the effect of a high tackle early in the game and whether it ‘sets the threshold’ for how subsequent tackles are judged. MacMahon, Starkes and Deakin^16^ found that information priming (eg, rules tests or foul videos) did not significantly improve basketball referees’ infraction detection. Instead, detection accuracy depended on the challenge and sequencing of video clips, emphasising the need for well-designed training tools that address the complexity of referee decision-making.

To maximise the effectiveness of the updated tackle height law (prevention practice), it is important to understand whether referees consistently enforce the law. Therefore, this study aimed to understand whether individuals who are refereeing Rugby matches can independently reach the same conclusion in a controlled environment (ie, video) regarding the high tackle law. The study also examined whether exposure to examples of dangerous or legal tackles during training for the study influenced decisions in a controlled video environment.

## Methods

### Study design and participant recruitment

This study followed a cross-sectional study design. A series of questions aligned with each step of the high tackle framework provided by World Rugby^8^ and a video compilation of pre-coded tackles were securely distributed to referees (active within the previous 12 months) on REDCap^17 18^ and Microsoft SharePoint Online, respectively. The study was advertised through social media and referee networks (Oct 2023–Feb 2024). Interested referees emailed the lead researcher to express their interest. Participants were blinded to the study’s primary purpose (their ability to interpret the trial high tackle law consistently with one another) and were instead informed it was to gain an understanding of the application of the change in law. Ethics approval was obtained from the Research Ethics Approval Committee for Health at the University of Bath (MSES 22/23–015). All participants were provided with informed consent before completing the questionnaire.

Participants received instructional videos, including an example tackle from the same participation level (Boys’ School Rugby). Participants were randomly assigned either an illegal (high) or legal example tackle tutorial to investigate the effects of priming (ie, whether seeing a dangerous high tackle influences future decision-making). Before watching the tackles, participants were asked to complete a demographics questionnaire to record information regarding sex, nationality and their refereeing experience, including highest level refereed, most frequent level refereed and years refereeing (online supplemental appendix B). Participants were then asked to complete the questionnaire for each tackle in the test set (online supplemental appendix C). The questionnaire aligned with the existing World Rugby high tackle decision-making flow diagram and were face validated for use in a community rugby setting by an experienced former referee (14 years of experience), an experienced coder (5 years of experience) and three research team members to ensure relevancy.^8 19^

### Video selection

The test set included eighteen tackles selected from previously coded, publicly available boys English School Rugby matches. Fifteen were unique tackles, and three were repeated to measure intra-rater reliability. The set included high tackles of varying degrees of danger and legal tackles to act as a control. Videos were recorded from level with the halfway line on one side of the pitch using one camera.

### Data analysis

All statistical analyses were conducted using Stata 18.^20^ Participants were only included if they completed the assessment of at least one tackle. In seven cases where participants duplicated assessments of the same tackle, only the final attempt was retained for analysis. The first assessments were retained in two cases where assessments were repeated for the entire test set. Participants’ qualification levels and highest/most frequent levels of refereeing were categorised: Local Societies, Regional Level, National League Level, and Professional Game Match Official Team.^21^

Descriptive statistics were calculated and reported as frequencies and means (95% CIs) or medians and IQRs where the data was non-normally distributed. Response accuracy was expressed as a percentage agreement between the experienced coding analyst and the experienced referee and with other responding referees. Inter-rater and intra-rater reliability were assessed using percent agreement and Gwet’s Agreement Coefficient (AC).^22 23^ Interpretations of Gwet’s AC were following those reported by Landis and Koch (online supplemental appendix D).^24^

### Patient and public involvement statement

A referee and other stakeholders (two referee educators) were engaged in developing the study questions and methodology. Based on feedback from these groups, the methodology was refined to be more user-friendly and robust. One referee was a research team member, and the other stakeholders were members of the wider rugby community. No other patients or members of the public were involved.

## Results

Of 79 participants who completed the demographics survey (online supplemental appendix B), 31 did not complete any tackle assessments, leaving a final sample of 48 (51.6%) referees for analyses. Of these, 30 (62.5%) received the legal tackle tutorial, and 19 (39.6%) received the high tackle tutorial. Forty-four participants (91.7%) indicated they were refereeing in a country implementing the law trial change, which set the maximum tackle height at the base of the sternum. Participant demographics can be found in table 1.

**Table 1:** Participant demographics

Participant characteristics	Summary (n=48 referees)	
Age, median (IQR)	47 years	(18 years)
Biological sex, n (%)	Male: 45	(93.8%)
	Female: 3	(6.3%)
Countries, n (%)	UK: 30	(62.5%)
	Ireland: 7	(14.6%)
	South Africa: 4	(8.3%)
	Germany: 2	(4.2%)
	USA: 2	(4.2%)
	Belgium: 1	(2.1%)
	France: 1	(2.1%)
	Mexico: 1	(2.1%)
Years refereeing, median (IQR)	7.5 years	(9.5 years)
Qualifications, n (%)	None: 3	(6.3%)
	Local societies: 35	(72.9%)
	Regional level: 1	(2.1%)
	National League level: 5	(10.4%)
	Professional/international: 4	(8.3%)
Highest level, n (%)	Local societies: 26	(54.2%)
	Regional level: 11	(22.9%)
	National level: 8	(16.7%)
	Professional/international: 3	(6.3%)
Most frequent level, n (%)	Local societies: 33	(68.8%)
	Regional level: 8	(16.7%)
	National level: 6	(12.5%)
	Professional/international: 1	(2.1 %)

In total, 801 of a possible 864 (92.7%) tackles were assessed (online supplemental appendix E; median=18 tackles per referee, range: 1–18). Participants were instructed to provide an initial response when watching the tackle at normal speed before slowing the clip down and rewatching. After watching the clip at a slower speed, 8.0% (n=64) opted to change their response, the most common change being from legal to high (7.7%, n=62).

The chance adjusted inter-rater agreement regarding tackle height at a slow speed was fair between participants (Gwet’s AC: 0.39 (0.23–0.55)), moderate between participants and the gold standard referee (Gwet’s AC: 0.51 (0.32–0.70)), and fair between the participants and analyst (Gwet’s AC: 0.46 (0.25–0.67)) (table 2). When a high tackle was reported between participants, agreement was substantial regarding the degree of danger (Gwet’s AC: 0.61 (0.43–0.79)) and fair for the questions regarding penalty level (Gwet’s AC: 0.35 (0.55–0.72)) and whether mitigating factors were present (Gwet’s AC: 0.21 (0.07–0.35)). Participants also showed moderate agreement with the gold standard referee on the degree of danger (Gwet’s AC: 0.56 (0.36–0.76)) and previous guidelines (Gwet’s AC: 0.57 (0.35–0.79)) and fair agreement on penalty level (Gwet’s AC: 0.36 (0.14–0.57)) and the presence of mitigating factors (Gwet’s AC: 0.24 (0.03–0.46)). Analyst-gold standard referee agreement regarding tackle height was moderate (72.2%; percent agreement 0.72, 95% CI 0.49 to 0.95; Gwet’s AC 0.52, 95% CI 0.06 to 0.97).

**Table 2:** The inter-rater agreement between all participants and between participants and the gold standard measures (analyst and referee) on their decision regarding whether a tackle was high or not

Item	Percentagreement (%)	Overall agreement			Agreement with gold standard referee			Agreement with analyst	
		Percent AC	Gwet’s AC	Percentagreement (%)	Percent AC	Gwet’s AC	Percentagreement (%)	Percent AC	Gwet’s AC
Height	78.1	0.70(0.62–0.78)	0.39(0.23–0.55)	74.0	0.70(0.62–0.78)	0.51(0.32–0.70)	62.7	0.70(0.62–0.78)	0.46(0.25–0.67)
Degree of danger	82.6	0.72(0.62–0.82)	0.61(0.43–0.79)	73.3	0.71(0.63–0.80)	0.56(0.36–0.76)			
Penalty	62.0	0.48(0.39–0.56)	0.55(0.35–0.72)	60.5	0.63(41.9–88.6)	0.36(0.14–0.57)			
Mitigating factors	70.8	0.60(0.54–0.67)	0.21(0.07–0.35)	43.4	0.61(0.53–0.69)	0.24(0.03–0.46)			
Old guidelines	81.6	0.72(0.63–0.82)	0.53(0.31–0.75)	77.8	0.72(0.63–0.82)	0.57(0.35–0.79)			

Actual percent (%), percent agreement coefficients (Ppercent AC), and Gwet’s AC coefficients (95% CI) are reported.

Footnote;: Sshading corresponds with agreement coefficient interpretation by Landis and Koch (1977),^24^ that is, the darker the box the greater the agreement.

ACAgreement Coefficient

For intra-rater reliability, mean agreement was 91.2% (Gwet’s AC: moderate–0.85 (0.52–1.18)) with a range between tackles of 84.6% to 100% agreement (table 3). Referees working at higher levels or local societies had similar reliability for assessing a high tackle (table 4). Exposure to a training video clip which was either legal or high did not significantly affect the proportion of tackles which were subsequently reported as high (table 5).

**Table 3:** Overall intra-rater reliability for the three repeated tackles: percent agreement, percent agreement coefficient and Gwet’s AC (95% CI)

Repeated tackle pair	Height (yes/ no)		
	Agreement (%)	Percent agreement coefficient	Gwet’s AC
1	100.0	1.00	1.00
2	84.6	0.85 (0.73–0.96)	0.74 (0.53–0.96)
3	88.9	0.89 (0.78–1.00)	0.81 (0.61–1.00)
Mean	91.2 (71.4–100.0)	0.92 (0.73–1.00)	0.85 (0.52–1.00)

ACAgreement Coefficient

**Table 4:** Agreement by demographic (highest level refereed: local societies vs higher) on whether a tackle was high, reported as median (range)

Highest level refereed	N (%)	Responses	% Yes	Percent agreement	Gwet’s AC
Local societies	26 (54.2%)	415	216 (52.1%)	0.71 (0.58–0.83)	0.43 (0.16–0.70)
Regional, national, professional	22 (45.8%)	368	197 (53.5%)	0.70 (0.56–0.84)	0.41 (0.07–0.75)

ACAgreement Coefficient

**Table 5:** Effects of priming on whether participants reported a tackle as high; median (range)

Tutorial group	N (%)	% Yes
Legal	19 (39.6)	66.7 (0.0–100.0)
High	29 (60.4)	56.9 (0.0–94.7)

**Figure 1:**
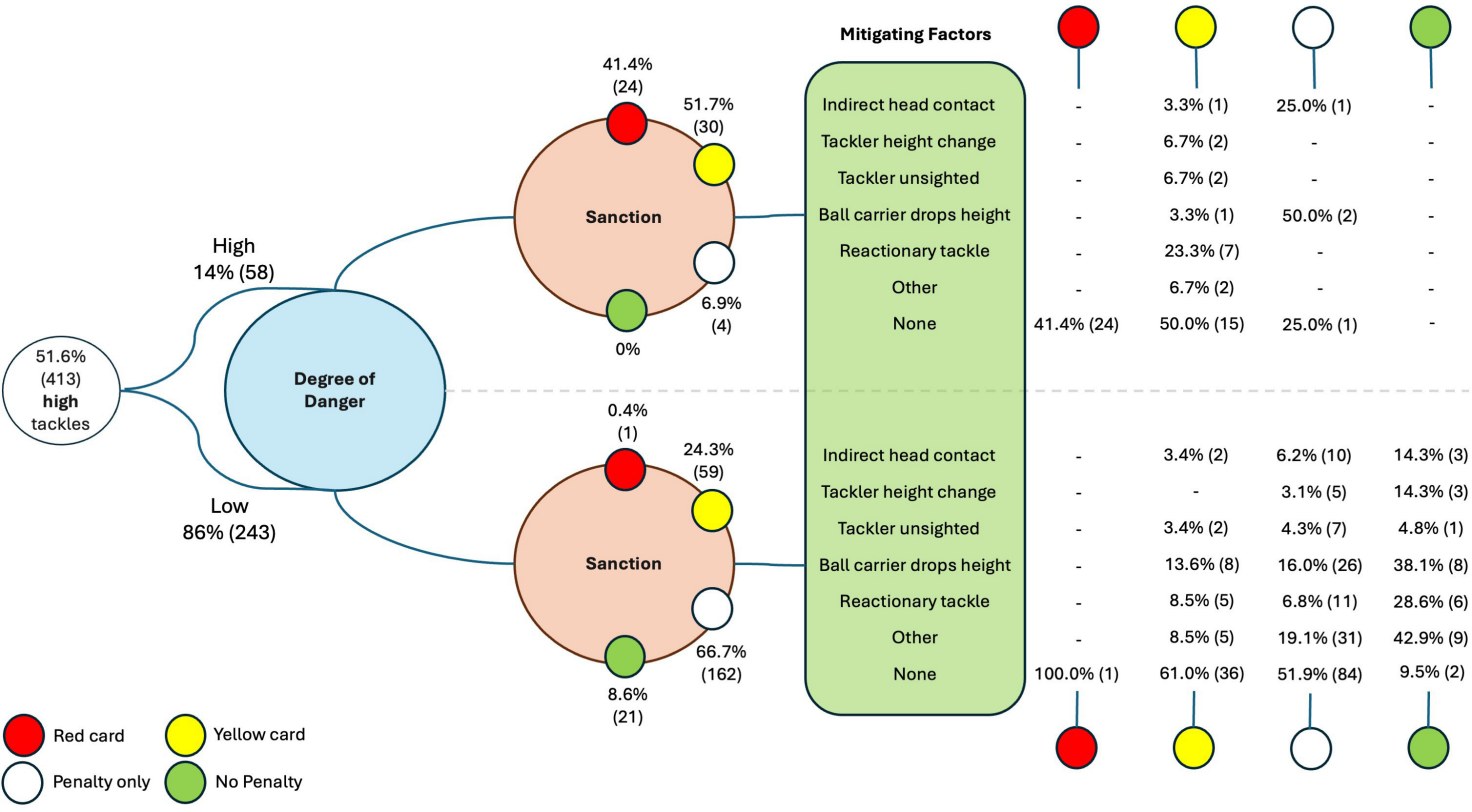
The frequency and percentage of responses relative to total responses for each outcome in the preceding question, illustrated in a decision-making pathway diagram depicting the probability of answers across three sequential questions: degree of danger, sanction associated with each tackle and the mitigating factors present. IHC, indirect head contact; CH, tackler makes definite attempt to change height; US, tackler unsighted prior to contact; BC, ball carrier suddenly drops in height; RT, ‘reactionary tackle’-immediate release.

Figure 1 illustrates how participants who identified a tackle as high subsequently assigned a level of danger, determined the appropriate sanction and indicated any mitigating circumstances, if present. All participants who determined the appropriate sanction for a tackle (eg, red card) also identified no mitigating factors (figure 1). However, one participant assessed a tackle as having a high degree of danger with no mitigating factors but still determined that only a penalty kick, rather than a more severe sanction, was appropriate.

## Discussion

This study has shown fair to moderate levels of agreement between referees, between referees and a gold standard referee and between referees and a video analyst. Having, at most, moderate agreement among referees for identifying and correctly assessing a high tackle suggests this may translate into inconsistent enforcement of the rules. This may cause confusion, undermine the law’s credibility and lead to disputes, making it difficult to ensure safety and fairness across games. Moreover, our findings of moderate agreement (72.2%) between referees and research video analysts support a need for greater involvement of referees within injury prevention and tackle law research to ensure the laws are practical and can be consistently applied. Finally, our pilot investigation into the effect of priming before video assessments showed no differences in the referees’ decision-making.

### Referee agreement

With an accuracy of only 67.2% (fair agreement with analyst), the findings suggest that many high tackles—without accounting for contextual factors or referee discretion—are being missed in matches. This weakens the message to players that there is zero tolerance for head contact and consequently may impact the effectiveness of injury prevention and player safety initiatives.^7^

Moderate agreement between the experienced analyst and gold standard referee may suggest the importance of understanding and considering experiential influences. This is also supported by a higher agreement between participants and the gold standard referee (moderate) than between participants and analysts. Previous studies in other sports, including youth basketball^25^ and ice hockey,^26^ have shown a low rate of penalisation for head contact in sports. This implies that rule enforcement may be lenient, and players’ experiences may not align with the intended strictness of these rules. This may be particularly relevant for participants who reported the youth rugby context as a mitigating factor. This suggests a more lenient approach, favouring an increased flow of the game at the expense of the intended safety aspects of such rules. As such, it is important to focus on strengthening the enforcement of the tackle height law alongside its implementation.

The inter-rater agreement between participants regarding the degree of danger was substantial but only moderate when compared with the gold standard referee. With the consequences of incorrect decisions likely dangerous (ie, may result in head injury), it is perhaps more suitable to accept a minimum of 80% inter-rater agreement as sufficient, which is recommended in most clinical research literature.^27^ Our findings of agreement below this threshold demonstrate the need for measures to improve referee decision-making. Moreover, some participants acknowledged the presence of mitigating factors but advocated for different levels of penalty. There was also little agreement on mitigating factors for each tackle (when applicable). This may suggest increased ambiguity and subjectivity in the interpretation of the laws, causing inconsistency in law application.

Across all tackles, the between-participant inter-rater agreement was greater than the agreement with the experienced analyst or gold standard referee. In addition, Gwet’s AC (0.52, 95% CI 0.06 to 0.97) suggests only a slight agreement between the experienced coder and the gold standard referee. This may be explained by the experienced analyst’s lack of ‘real-world’ refereeing experience. The analyst may have taken a more objective approach to determining whether a tackle was high, while the referees may have considered additional environmental factors (eg, many participants reported the youth Rugby context as a mitigating factor). Therefore, future research should involve experienced field professionals as coders or include them in the analyst training process.

### Priming

Given the exploratory nature of this secondary objective and the small, unequal sample sizes between tutorial video groups, the absence of priming should be interpreted with caution. It is possible that referees were not swayed by exposure to a single, particularly dangerous high tackle, instead evaluating each tackle on its own merits. However, the lack of evidence for priming may also suggest that referees could benefit from increased vigilance in identifying and penalising such infractions during subsequent tackles. This could contribute to underreporting high tackles and an increased risk of injury, posing a concern for player welfare. Raab and colleagues^28^ proposed a threshold model for decision-making in sport, suggesting that individuals may have different thresholds for how much a change in context (eg, increased aggression) influences game management behaviours, which could affect rule enforcement. This may indicate that the effects of priming are more likely to emerge during live match-play for most referees.

An almost perfect overall intra-rater reliability may suggest that referees are consistent in decision-making. While this is promising, it could become an issue if a referee consistently makes an incorrect decision. Given that these decisions were made in a controlled environment (ie, free from environmental factors including crowds, players, and in-game pressure and fatigue), this may suggest that any biases on the referee’s part are implicit and, hence, cannot easily be changed.^29^ It may also suggest the importance of TV match officials to provide more consistent decision-making in a controlled environment, as it is likely that decisions are influenced during match-play when they are fatigued and have external distractions.^12 13^ However, this does not apply to the community/grassroots game. It is vital that individuals demonstrate an understanding of the laws and how to enforce them before being permitted to referee Rugby matches.^11^

Training videos are used to teach and practice tackle techniques in a controlled environment (eg, Tackle Ready; World Rugby Passport).^30^ However, this approach primarily targets players and coaches, raising whether referees fully use this resource. Moreover, continuing to include videos that clearly distinguish between the two tackle height laws and examples of the mitigating factors in each tackle may enhance this process. Additionally, using existing resources in the law implementation website provided by World Rugby, which includes clips from the referee’s vantage point, could be beneficial. This video analysis and training may support more effective law implementation, hence greater widespread injury prevention.^10 11^

### Limitations

Referees were first prompted to watch tackles at full speed to assess decision differences between real-time and slowed-down viewing; however, participants may have viewed clips several times, influencing reliability. Future research should assess decision-making under lab-controlled conditions to ensure strict adherence to protocol. This may also affect the generalisability of the findings. Controlling the footage speed helped to explore the interpretation of the tackle height law, but this would not translate to a match situation. The camera angles were also inconsistent with actual referee positioning during a match. Future research could consider using point-of-view cameras affixed to referees to mitigate the limitations posed by camera angles, as the angle of view can directly impact the interpretation of key factors, such as the point of contact. This approach would help assess whether referees can accurately judge tackle height during match play, where most injuries occur,^31^ and could open up further research questions on how viewing perspective influences decision-making. School-level match footage may limit the generalisability, as many referees cited this as a mitigating factor. However, since the updated trial law is currently applied only in community and youth Rugby, assessing decision-making across different levels of play could provide valuable insights into how this affects referees’ decisions. Regarding the priming investigation, this was limited by not being able to ensure participants watched the tutorial video and differences in group sizes.

## Conclusion

This study highlights several considerations regarding referee agreement on tackle height laws. There is considerable variability in the assessment of tackle height and appropriate sanction. Further, inter-rater agreement among participants and with the gold standard referee was below the recommended 80% clinical threshold, suggesting a high likelihood of inconsistencies in implementing tackle height laws. This may undermine the zero-tolerance policy for head contact. This study underscores the urgent need to enhance training resources and incorporate simulations that account for experiential factors, and advocates for revising existing referee training models to align with the updated laws.

## supplementary material

10.1136/bmjsem-2024-002347 online supplemental file 1

## Data Availability

No data are available.
