# Supplementary material for: High tackle headache: implications of referee agreement for tackle height law change
Source: BMJ Open Sport Exerc Med. 2025 Jan 20;11(1):e002347. doi: 10.1136/bmjsem-2024-002347 (PMC11781086; doi:10.1136/bmjsem-2024-002347)
Supplement: online supplemental file 1 [file bmjsem-11-1-s001.docx]

**Appendix A**

**Video signs indicating higher degree of danger (World Rugby, 2021):**

- Tackler draws the arm back prior to contact.
- Tackler may leave the ground.
- Arm swings forward prior to contact.

**Contact:**

- Tackler is attempting an active/dominant tackle, as opposed to passive/soak, or “pulling out” of contact.
- Tackler speed and/or acceleration into tackle is high.
- Rigid arm or elbow makes contact with ball carrier (BC) head as part of a swinging motion.

**Follow Through:**

- Tackler completes the tackle (as opposed to immediate release/withdrawal).

**Factors to consider against mitigation:**

- If the tackler and BC are in open space and/or the tackle has clear line of sight and time before contact.

**Mitigating factors:**

(Must be clear and obvious and can only be applied to reduce a sanction by one level)

- Tackler makes a definite attempt to change height in an effort to avoid ball carrier’s head.
- BC suddenly drops in height (e.g., from earlier tackle, trips/falls, dives to score).
- Tackler is unsighted prior to contact.

“Reactionary” tackle, immediate release.

**Appendix B**

Refereeing experience questionnaire:

- 1. *Please enter your age in years*
  2. *Please enter your biological sex*
  3. *How many years have you been refereeing? Please include every year you have refereed at least one match.*
  4. *Do you hold a current referee qualification?*
     - 1. *If yes, what qualification do you hold?*
  5. *In what country have you predominantly refereed in in the previous 12 months?*
  6. *Has the tackle height law lowered in your country as of this season? For example, in community Rugby in England, the tackle height law has been lowered from the line of the shoulder to the base of the sternum.*
  7. *What is the highest level of Rugby you have refereed at?*
  8. *What is the most frequent level of Rugby you referee?*
  9. *Which sex do you referee most often?*

**Appendix C**

**Tackle Height Questionnaire**

The questionnaire included three online forms: consent, demographics and refereeing experience (Appendix 3), and the tackle height questionnaire. The tackle height questionnaire included the following:

*Please answer the following questions as though you are refereeing with the lowered tackle height law in place (tackles must be below the base of the sternum).*

*Please watch the video at full speed…*

1. *Is it a high tackle? (Yes/ No)*

*Please now rewatch the clip as many times as you wish, using the slow-motion feature or pausing when required…*

1. *Is it a high tackle? (Yes/ No)*
   1. *What part of the tackler’s body made the high contact? (Shoulder/ Head/ Arm/ Other)*
      1. *If the tackler made contact with their shoulder or head, was the degree of danger high or low?*
      2. *If the contact was made by the tackler’s arm, did the arm make contact with the head/ neck of the ball carrier? If yes, was the degree of danger high or low?*
   2. *What level of penalty would you associate with this tackle? (Red Card and Penalty Kick/ Yellow Card and Penalty Kick/ Penalty Kick Only/ None- Play On)*
   3. *Were there any mitigating factors? (Yes/ No)*
      1. *If yes, what were the mitigating factors present?*

*Under the previous tackle height guidelines which were in place until the end of the 2022/23 season, would you penalise this tackle*

**Appendix D**

Benchmark scale for agreement coefficient agreement point interpretation by Landis and Koch (1977)

| Coefficient | Interpretation |
| --- | --- |
| Below 0.00 | Poor |
| 0.00 – 0.20 | Slight |
| 0.21 – 0.40 | Fair |
| 0.41 – 0.60 | Moderate |
| 0.61 – 0.80 | Substantial |
| 0.81 – 1.00 | Almost Perfect |

**Appendix E**

The frequency of each response of key questions given as n (%), where n denotes the frequency and % denotes the percentage of total responses (including the gold standard referee) for that question (RC denotes red card and penalty kick; YC, yellow card and penalty kick; PK, penalty kick only; none, no penalty- play on; N/A indicates that no participants answered a follow-up question; and ^a b c^ indicate the pairs of repeated tackles).

The highest percent agreement for each question for each tackle is highlighted based on its respective Cohen’s Kappa level of agreement (McHugh, 2012).

Key:

Almost Perfect

Strong

Moderate

| Tackle Number | Full-Speed- High? | | Adjusted-Speed- High? | | Danger | | Penalty | | | | Any mitigating factors? | | Old Guidelines- Penalise? | |
| --- | --- | --- | --- | --- | --- | --- | --- | --- | --- | --- | --- | --- | --- | --- |
|  | Yes | No | Yes | No | High | Low | RC | YC | PK | None | Yes | No | Yes | No |
| 1 | 31 (67.4%) | 15 (32.6%) | 35 (76.1%) | 11 (23.9%) | 0  (0.0%) | 13 (100.0%) | 0 (0.0%) | 1 (2.9%) | 31 (88.6%) | 3 (8.6%) | 11 (31.4%) | 24 (68.6%) | 21 (45.7%) | 25 (54.3%) |
| 2 | 44 (93.6%) | 3  (6.4%) | 44 (93.6%) | 3  (6.4%) | 14 (34.1%) | 27 (65.9%) | 3 (6.8%) | 21 (47.7%) | 19 (43.2%) | 1 (2.3%) | 23 (52.3%) | 21 (47.7%) | 40 (85.1%) | 7 (14.9%) |
| 4 ^a^ | 0 (0.0%) | 44 (100.0%) | 0  (0.0%) | 44 (100.0%) | N/A | N/A | N/A | N/A | N/A | N/A | N/A | N/A | 0 (0.0%) | 44 (100.0%) |
| 7 ^b^ | 28 (60.9%) | 18 (39.1%) | 31 (67.4%) | 15 (32.6%) | 6  (19.4%) | 25 (80.6%) | 6 (19.4%) | 11 (35.5%) | 13 (41.9%) | 1 (3.2%) | 11 (35.5%) | 20 (64.5%) | 21 (45.7%) | 25 (54.3%) |
| 9 | 33 (73.3%) | 12 (26.7%) | 38 (84.4%) | 7 (15.6%) | 2  (11.1%) | 16 (88.9%) | 1 (2.6%) | 6 (15.8%) | 28 (73.7%) | 3 (7.9%) | 16 (42.1%) | 22 (57.9%) | 12 (26.7%) | 33 (71.7%) |
| 13 | 15 (33.3%) | 30 (66.7%) | 18 (40.0%) | 27 (60.0%) | 1  (20.0% | 4 (80.0%) | 0 (0.0%) | 1 (5.6%) | 15 (83.3%) | 2 (11.1%) | 2 (11.1%) | 16 (88.9%) | 2 (4.4%) | 43 (95.6%) |
| 17 | 27 (58.7%) | 19 (41.3%) | 32 (69.6%) | 14 (30.4%) | 2  (6.9%) | 27 (93.1%) | 1 (3.1%) | 4 (12.5%) | 25 (78.1%) | 2 (6.3%) | 19 (59.4%) | 13 (40.6%) | 21 (45.7%) | 25 (54.3%) |
| 18 | 5 (11.1%) | 40 (88.9%) | 6 (13.3%) | 39 (86.7%) | 1  (25.0%) | 3 (75.0%) | 0 (0.0%) | 2 (33.3%) | 3 (50.0%) | 1 (16.7%) | 5 (83.3%) | 1 (16.7%) | 4 (8.9%) | 41 (91.1%) |
| 24 ^a^ | 0 (0.0%) | 45 (100.0%) | 0  (0.0%) | 45 (100.0%) | N/A | N/A | N/A | N/A | N/A | N/A | N/A | N/A | 0 (0.0%) | 45 (100.0%) |
| 27 | 8 (17.8%) | 37 (82.2%) | 11 (24.4%) | 34 (75.6%) | 0  (0.0%) | 8 (100%) | 0 (0.0%) | 1 (9.1%) | 4 (36.4%) | 6 (54.5%) | 10 (90.9%) | 1 (9.1%) | 4 (8.9%) | 41 (91.1%) |
| 28 | 9 (20.4%) | 35 (79.6%) | 11 (25.0%) | 33 (75.0%) | 0  (0.0%) | 2  (100%) | 0 (0.0%) | 2 (18.2%) | 4 (36.4%) | 5 (45.4%) | 9 (81.8%) | 2 (18.2%) | 3 (6.8%) | 41 (93.2%) |
| 29 ^c^ | 22 (51.2%) | 21 (48.8%) | 31 (72.1%) | 12 (27.9%) | 4  (16.7%) | 20 (83.3%) | 2 (6.5%) | 6 (19.4%) | 17 (54.8%) | 6 (19.4%) | 11 (35.5%) | 20 (64.5%) | 15 (34.9%) | 28 (65.1%) |
| 31 | 9 (20.4%) | 35 (79.6%) | 14 (31.8%) | 30 (68.2%) | 3  (27.3%) | 8 (72.7%) | 1 (7.1%) | 2 (14.3%) | 7 (50.0%) | 4 (28.6%) | 11 (78.6%) | 3 (21.4%) | 7 (15.9%) | 37 (84.1%) |
| 32 ^b^ | 25 (65.8%) | 13 (34.2%) | 29 (76.3%) | 9 (23.7%) | 4  (14.3%) | 24 (85.7%) | 3 (10.3%) | 11 (37.9%) | 15 (51.7%) | 0 (0.0%) | 13 (44.8%) | 16 (55.2%) | 20 (52.6%) | 18 (47.4%) |
| 33 ^c^ | 20 (55.6%) | 16 (44.4%) | 24 (66.7%) | 12 (33.3%) | 2  (11.1%) | 16 (88.9%) | 1 (4.2%) | 5 (20.8%) | 15 (62.5%) | 3 (12.5%) | 10 (41.7%) | 14 (58.3%) | 13 (36.1%) | 23 (63.9%) |
| 34 | 37 (90.2%) | 4  (9.8%) | 40 (97.6%) | 1  (2.4%) | 12 (32.4%) | 25 (67.6%) | 3 (7.5%) | 17 (42.5%) | 20 (50.0%) | 0 (0.0%) | 19 (47.5%) | 21 (52.5%) | 38 (92.7%) | 3  (7.3%) |
| 35 | 9 (21.4%) | 33 (78.6%) | 13 (30.9%) | 29 (69.1%) | 3  (42.9%) | 4 (57.1%) | 1 (7.7%) | 1 (7.7%) | 10 (76.9%) | 1 (7.7%) | 2 (15.4%) | 11 (84.6%) | 3 (7.1%) | 39 (92.9%) |
| 36 | 31 (75.6%) | 10 (24.4%) | 36 (87.8%) | 5 (12.2%) | 4  (16.7%) | 20 (83.3%) | 3 (8.3%) | 2 (5.6%) | 30 (83.3%) | 1 (2.8%) | 3 (8.3%) | 33 (91.7%) | 6 (14.6%) | 35 (85.4%) |
